# Polysaccharides from *Atractylodes macrocephala*: A Review of Mechanistic and Therapeutic Insights into Intestinal Disorders

**DOI:** 10.3390/nu17233722

**Published:** 2025-11-27

**Authors:** Meng Li, Chester Yan Jie Ng, Huangyan Chen, Wai Ching Lam, Linda Zhong

**Affiliations:** 1 School of Biological Sciences, Nanyang Technological University, 60 Nanyang Drive, Singapore 637551, Singapore; mengli@ntu.edu.sg (M.L.); yanjiech001@e.ntu.edu.sg (C.Y.J.N.); huangyan001@e.ntu.edu.sg (H.C.); waiching001@e.ntu.edu.sg (W.C.L.); 2 Department of Epidemiology, Harvard T.H. Chan School of Public Health, 677 Huntington Avenue, Boston, MA 02115, USA

**Keywords:** *Atractylodes macrocephala*, polysaccharides, intestinal inflammation, gut microbiota, immune modulation, gut–brain axis

## Abstract

Intestinal health is essential for maintaining systemic physiological balance through nutrient absorption, immune regulation, and host–microbiota interactions. *Atractylodes macrocephala* (Baizhu), a traditional medicinal plant long used for gastrointestinal dysfunction, has attracted growing interest because its polysaccharides (AMPs) show promises in intestinal disorders. In this review, we summarize preclinical studies on AMPs identified through searches of PubMed, Web of Science, ScienceDirect, Google Scholar and the China National Knowledge Infrastructure (CNKI), focusing on their extraction, purification, structural features and gut-related activities. Experimental evidence suggests that AMPs are metabolized by gut microbiota into short-chain fatty acids and other bioactive metabolites that regulate mucosal immunity, enhance epithelial barrier function and modulate host metabolic pathways. AMPs have been shown to promote the growth of beneficial taxa, restore dysbiotic communities, up-regulate tight junction proteins, suppress intestinal inflammation and modulate gut–brain axis signaling involved in intestinal motility and visceral sensitivity. These actions underlie their protective effects reported in models of irritable bowel syndrome (IBS), inflammatory bowel disease (IBD), colorectal cancer, chemotherapy-induced mucosal injury, and metabolic-associated systemic inflammation. Overall, current data support AMPs as microbiota-modulating, immunomodulatory, epithelial-protective and neuro-regulatory agents with potential as functional food-derived interventions for intestinal health. In this review, we also highlight key limitations and priorities for future research on structure–function relationships and clinical translation.

## 1. Introduction

The intestine serves as a major interface between the host and the external environment, integrating nutrient digestion and absorption, immune surveillance, and microbial metabolism [1]. This complex organ harbors an estimated 10^14^ microorganisms whose metabolic products continuously interact with epithelial and immune cells to maintain homeostasis [2]. Disruption of this equilibrium—characterized by dysbiosis, increased permeability, and chronic inflammation—underlies the pathogenesis of intestinal disorders such as irritable bowel syndrome (IBS), inflammatory bowel disease (IBD, encompassing ulcerative colitis and Crohn’s disease), and colorectal cancer (CRC) [3,4,5]. Beyond the intestine, impaired barrier integrity and translocation of microbial components trigger systemic inflammation and metabolic dysregulation, contributing to obesity, insulin resistance, and metabolic dysfunction-associated steatotic liver disease (MASLD) [6,7]. Current therapeutic options, including corticosteroids, biologics, and probiotics, can transiently relieve symptoms or inflammation but rarely restore durable intestinal homeostasis [8,9]. These limitations underscore the need for natural, safe, and mechanistically defined agents capable of rebuilding intestinal integrity and symbiotic microbial metabolism. Among such agents, polysaccharides derived from edible and medicinal plants—such as *Atractylodes macrocephala* Koidz. (Bai Zhu)—have emerged as promising candidates for the integrated modulation of gut health [10,11].

Natural polysaccharides represent a structurally diverse class of macromolecules that exert multifaceted effects on gut physiology. Most resist digestion in the upper gastrointestinal tract and are fermented by commensal microbiota to yield bioactive metabolites such as short-chain fatty acids (SCFAs), indole derivatives, and secondary bile acids, which influence barrier integrity, mucosal immunity, and host metabolism [12]. Classical dietary fibers like inulin and pectin primarily act as substrates that enrich butyrate-producing bacteria and reinforce barrier integrity [13]. In contrast, polysaccharides from medicinal plants exhibit more intricate structure–function relationships, characterized by branched architectures, uronic acid substitutions, and specific glycosidic linkages that enable selective microbial utilization and direct immunomodulation [14,15,16]. This deeper level of host–microbe crosstalk has drawn attention to medicinal polysaccharides as a distinct subclass of functional glycans that bridge nutrition and pharmacology within the food–medicine continuum [17,18]. Among these, polysaccharides from *A. macrocephala* (AMPs) provide an illustrative model for elucidating how structural diversity translates into targeted modulation of gut microbiota, immune responses, and epithelial homeostasis [19,20].

*A. macrocephala* is a widely used medicinal and dietary herb in traditional Chinese medicine. It is traditionally prescribed to “strengthen the spleen” and “dry dampness” and is commonly used to relieve gastrointestinal dysfunctions such as abdominal distension, diarrhea, and poor appetite, which broadly correspond to chronic functional bowel disturbances in modern clinical terms [21,22]. Pharmacological studies in preclinical models have reported that extracts of *A. macrocephala*, in which polysaccharides are major constituents, and particularly purified polysaccharide fractions exert anti-inflammatory, antioxidant, immunoregulatory effects and gastrointestinal-protective effects while promoting microbial balance and epithelial repair [23]. AMPs are heterogeneous macromolecules with β-glucan- and pectic-type backbones, whose structural diversity contributes to distinct physicochemical properties, microbial utilization patterns and biological activities [24]. On the basis of these findings, current evidence supports AMPs as gut-targeted candidates that may influence microbial metabolism, mucosal immunity and barrier function in the context of intestinal health.

Despite increasing research on AMPs, systematic integration of chemical, microbiological, and functional data remains limited. This review synthesizes recent advances linking the chemical diversity of AMPs to their gut-targeted biological functions. It first outlines current extraction and purification methods and summarizes the structural characteristics of AMPs—including monosaccharide composition, molecular weight, and glycosidic linkages—that have been associated with their reported biological activities [25,26]. The subsequent sections integrate evidence from cellular and animal studies to illustrate the protective roles of AMPs in intestinal inflammation, barrier dysfunction, and metabolic disorders [27,28]. Mechanistic insights are highlighted, emphasizing microbiota-mediated and immunometabolic pathways that underpin their gut-targeted actions [28,29]. Finally, we discuss key challenges and research priorities, including standardization, multi-omics integration, and translational validation. By critically evaluating current evidence, this review aims to elucidate how AMPs act as complex microbiota-interacting glycans and inform their development as safe and effective interventions for intestinal health.

## 2. Extraction, Purification, and Structural Characterization of AMPs

Extraction efficiency strongly influences AMP yield and integrity. AMPs are widely recognized as bioactive macromolecules with complex structural diversity, whose physicochemical integrity depends on carefully controlled upstream processing. The extraction of AMPs typically begins with hot water decoction from dried rhizomes, followed by graded ethanol precipitation to obtain the crude polysaccharide fraction. This conventional approach remains widely used for its simplicity and scalability, but it suffers from limited yield, low selectivity, and potential degradation of labile glycosidic linkages, which may alter downstream compositional and bioactivity profiles [30,31,32]. In recent years, a spectrum of modern techniques—including ultrasound-assisted extraction (UAE), microwave-assisted extraction (MAE), enzyme-assisted extraction (EAE), and their hybrid approaches—have been developed to optimize the recovery of high-molecular-weight, functionally intact polysaccharides [23,32,33,34]. These strategies improve solubilization kinetics, reduce thermal degradation, and modulate glycosidic cleavage patterns [24,35]. UAE typically operates with shorter extraction times and lower bulk temperatures, and often in-creases AMP yields, but the associated acoustic cavitation can also alter chain-length distributions and higher-order conformations [23,36]. EAE reduces the temperature requirements of decoction-based processes and im-proves extraction efficiency by promoting cell-wall degradation, yet its performance depends strongly on the choice of enzyme cocktail and process control [33]. MAE can deliver high recoveries within minutes; however, the need for dedicated reactors currently limits its application in large-scale AMP production [37]. Taken together, these methodological differences may shape both the reported structures and the bioactivities of AMPs and should therefore be considered when comparing results across studies.

Purification is critical for obtaining relatively homogeneous AMP fractions suitable for structural analysis and bioactivity testing. Once extracted, multi-step purification procedures are employed to remove impurities and isolate polysaccharide-enriched fractions. Standard workflows involve protein and pigment removal (e.g., Sevag method, trichloroacetic acid precipitation, activated carbon treatment), followed by chromatographic separation via DEAE-cellulose ion exchange and size-exclusion columns such as Sephadex G-100/G-200 [24,38]. Further refinement by ultrafiltration and dialysis enables molecular-weight stratification, providing a basis for exploring associations between macromolecular properties and biological activities [39,40]. Importantly, comparative work on polysaccharides from the genus *Atractylodes* has shown that different combinations of extraction conditions and purification schemes can bias molecular-weight distributions, monosaccharide ratios and glycosidic linkages, and that such methodological choices may partly underlie the variation in structural features and biological effects attributed to AMPs across studies [23].

Structural elucidation provides the chemical basis for understanding AMP bioactivity. Comprehensive data on extraction, purification, and structural characteristics of reported AMP fractions are compiled in Table 1, highlighting methodological variations and key compositional trends across studies. Depending on extraction conditions and purification stringency, AMP fractions exhibit broad molecular-weight distributions ranging from approximately 3 to 200 kDa. The structural complexity of AMPs is reflected in their heterogeneity of monosaccharide composition, glycosidic linkages, branching degrees, and higher-order conformation [11]. Monosaccharide profiling by HPLC, GC-MS, or ion chromatography consistently identifies glucose as the dominant component, accompanied by varying proportions of galactose, arabinose, rhamnose, mannose, xylose, and uronic acids. These compositional variations are accompanied by substantial differences in backbone architecture [25,26,28,41,42,43,44,45,46,47,48,49,50,51,52,53,54,55,56]. Methylation-GC-MS analysis and 1D/2D NMR analyses reveal representative β-(1 → 3)-glucans with (1 → 6)-linked side chains in neutral fractions and rhamnogalacturonan-I-like domains enriched in galacturonic acid in acidic fractions. Some studies have reported the presence of inulin-type fructans with β-(2 → 1)-linked fructose units, indicating compositional diversity even within a single botanical source [50,52]. Secondary-structure assessments using circular dichroism and Congo red assays suggest conformational variability among AMP fractions, ranging from random coils to triple-helix-like motifs depending on ionic conditions and molecular size [48,57]. Such conformational variability may influence solubility, stability, and potential bioactivity. Collectively, these insights provide a chemical foundation for linking structural attributes to biological functions and for establishing standardized AMP preparations suitable for mechanistic and translational studies. Emerging analytical platforms—including LC–MS-based glycomics, high-resolution NMR, and molecular-weight-resolved chromatography—are expected to further refine AMP structural elucidation and deepen our understanding of how chemical composition relates to biological function [58,59,60].

## 3. Effects of AMP on Gut Health

### 3.1. Modulation of Gut Microbiota and Metabolites

The gut microbiota represents a primary interface through which AMPs exert their physiological and therapeutic effects [63]. As non-starch polysaccharides, AMPs resist enzymatic digestion and absorption in the upper gastrointestinal tract and reach the colon largely intact, where they serve as fermentable substrates for commensal bacteria [28,64]. This fermentation promotes the growth of beneficial taxa, suppresses opportunistic pathogens, and generates a variety of bioactive metabolites—including SCFAs, tryptophan derivatives, and bile-acid metabolites—that collectively regulate immune homeostasis, epithelial barrier integrity, and host metabolism [44,65,66].

Evidence from multiple experimental models indicates that AMPs consistently shift the gut microbial ecosystem toward a eubiotic configuration. In dextran sulfate sodium (DSS)-induced colitis, AMP supplementation increased the relative abundance of butyrate-producing genera such as *Butyricicoccus* and *Lactobacillus* [44,67]. Similarly, AMP restored overall microbial richness and diversity, reduced *Desulfovibrio* and *Enterobacteriaceae*, and enriched beneficial taxa including *Faecalibaculum* and *Bifidobacterium* [68]. These patterns are reproducible in non-murine species—for example, AMP administration in LPS-induced intestinal injury in goslings enhanced microbial diversity and reduced pathogen-associated genera [69]. Comparable eubiotic trends were also observed in spleen-deficiency constipation and cyclophosphamide-induced gut injury models, where AMP promoted fermentative, SCFA-producing genera such as *Odoribacter*, *Bacteroides*, and *Prevotella*, while suppressing inflammation-associated species [70]. Collectively, these findings suggest that AMPs act as broad-spectrum microbiota modulators capable of restoring microbial homeostasis across both inflammatory and metabolic contexts.

A hallmark outcome of AMP-microbiota interaction is the stimulation of SCFA production, particularly acetate, propionate, and butyrate. These metabolites serve as critical signaling molecules that bridge microbial activity and host physiology. Butyrate, for instance, fuels colonocyte oxidative metabolism, promotes regulatory T-cell (Treg) differentiation through histone deacetylase inhibition and Foxp3 upregulation, and supports mucin biosynthesis and tight-junction assembly, thereby fortifying the epithelial barrier [71,72]. In a spleen-deficiency constipation model, AMP restored fecal SCFA concentrations concomitant with the enrichment of *Parabacteroides* and ameliorating DSS-induced ulcerative colitis [44]. Related studies indicate that AMP treatment enriches propionate- and butyrate-producing bacteria and increases luminal SCFA output, thereby reinforcing the microbiota–SCFA–host axis that contributes to intestinal and metabolic homeostasis.

Beyond SCFAs, AMPs profoundly affect amino-acid-derived and bile-acid-derived microbial metabolites. AMP treatment enriches tryptophan-metabolizing bacteria such as *Lactobacillus* and *Rothia*, elevating indole derivatives (e.g., indole-3-propionic acid, indole, tryptamine, tryptophol) that activate aryl hydrocarbon receptor (AhR) and pregnane X receptor (PXR) signaling pathways [73,74]. Activation of these nuclear receptors induces IL-22 and glucagon-like peptide-1 (GLP-1) expression, enhances mucosal defense, and suppresses NF-κB–driven inflammation. Antibiotic-depletion or fecal-microbiota-transplantation experiments further confirm that the protective effects of AMPs are microbiota dependent [74]. Although direct metabolomic evidence remains limited, AMP-induced microbial restructuring is associated with secondary modulation of bile acid metabolism. In DSS colitis, AMP normalized bile-acid biosynthetic pathways [44]; in spleen-deficiency constipation, changes in primary bile-acid pools correlated with symptom relief [75]. These observations suggest that the microbial–metabolite–receptor axis is a key effector pathway through which AMPs confer intestinal protection.

AMPs reshape the intestinal microbial ecosystem and its metabolite landscape, enriching beneficial bacteria and reinforcing metabolite-mediated communication with the host. Through the SCFA, tryptophan, and bile-acid axes, AMPs establish a microbiota-metabolite-host network that underlies their protective and regulatory functions in the gut. These microbial and metabolite shifts collectively illustrate the central role of AMP–microbiota interactions in maintaining intestinal homeostasis [44,73,76].

### 3.2. Intestinal Barrier Protection and Epithelial Repair

The intestinal epithelium forms the first physical and biochemical line of defense against luminal antigens, pathogens, and toxins. It consists of a single layer of epithelial cells sealed by tight junctions (TJs) and covered by a mucus layer secreted by goblet cells [77,78]. In intestinal disorders—including IBD, IBS, CRC, and drug- or diet-induced injury—this barrier is frequently compromised, leading to microbial translocation and chronic mucosal inflammation [79,80]. AMPs help protect and restore intestinal barrier integrity through coordinated reinforcement of tight junction complexes, stimulation of mucin production, and acceleration of epithelial restitution. Multiple in vivo and in vitro studies demonstrate that AMPs upregulate key TJ proteins—zonula occludens-1 (ZO-1), occludin, and claudins—thereby strengthening intercellular adhesion and reducing paracellular permeability. In DSS-induced colitis, AMP supplementation significantly restored Claudin-1 expression and mitigated epithelial erosion [68]. Comparable outcomes were observed in senile constipation and pyrotinib-induced diarrhea, where AMP upregulated TJ protein expression and normalized villus morphology [81,82]. In a cyclophosphamide-immunosuppressed chick model, AMP treatment markedly elevated ZO-1 and occludin levels, resulting in the recovery of transepithelial electrical resistance and improved mucosal morphology [67]. Mechanistic studies further revealed that AMP-induced long non-coding RNA ITSN1-OT1 sequesters phosphorylated STAT2 in injured intestinal epithelial cells (IECs), preventing TJ degradation and restoring barrier continuity [27]. Beyond direct structural restoration, AMPs also activate host defense pathways that promote epithelial renewal. In rats with high-fat/high-sugar diet–induced glycolipid metabolic disorder, AMP elevated microbial tryptophan metabolites, which activated the AhR and subsequently increased IL-22 expression—an interleukin known to induce epithelial antimicrobial peptides and tight-junction proteins [73]. Together, these results indicate that AMPs strengthen the epithelial barrier through both transcriptional regulation and post-translational stabilization of junctional proteins.

In addition to tight junction reinforcement, AMPs fortify the mucus layer that overlays the epithelium. The mucus—primarily composed of the glycoprotein MUC2—forms a physical and biochemical shield against microbial invasion [83]. AMP treatment has been shown to restore goblet-cell density and increase MUC2 expression across multiple models. In DSS-colitis and metabolic-disorder rats, AMP administration resulted in a thicker mucus layer and improved epithelial morphology [68,81,84]. Histological analyses confirmed a marked reduction in goblet-cell depletion and increased mucus secretion, correlating with decreased bacterial adherence to the mucosa.

Beyond preserving barrier structures, AMPs actively stimulate epithelial repair through trophic and metabolic pathways. One well- defined mechanism involves polyamine biosynthesis, which supports cytoskeletal remodeling and wound closure. In IEC-6 monolayers scratch assays, AMP treatment elevated intracellular spermidine and spermine levels, activated Kv1.1 potassium channels, and increased Ca^2+^ influx, collectively accelerating epithelial migration and closure; these effects were abolished by α-difluoromethylornithine (DFMO), an ornithine decarboxylase inhibitor [85]. In vivo, AMP treatment improved villus height, enhanced re-epithelialization, and reduced ulcerative lesions following chemically induced mucosal injury [67,85]. These reparative effects are further supported by AMP-induced activation of growth-factor pathways, including elevated expression of epidermal growth factor (EGF) and transforming growth factor-β1 (TGF-β1) [85]. Combined with their antioxidant and anti-inflammatory properties, these trophic mechanisms create a favorable microenvironment for mucosal healing. Histological evidence consistently reveals AMP-treated animals exhibiting preserved epithelial architecture, reduced neutrophil infiltration, and increased goblet-cell abundance [69], corroborating the functional restoration of barrier integrity at both molecular and tissue levels.

Collectively, AMPs safeguard intestinal barrier integrity by enhancing tight-junction assembly, stimulating mucin secretion, and promoting epithelial restitution through polyamine- and growth-factor–mediated pathways.

### 3.3. Bidirectional Immune Regulation and Anti-Inflammatory Mechanisms

The therapeutic efficacy of AMPs in intestinal disorders arises largely from their bidirectional immunoregulatory capacity—enhancing suppressed immunity while restraining excessive inflammation. Rather than acting as simple immunostimulants or suppressants, AMPs dynamically recalibrate both innate and adaptive immune responses to restore mucosal homeostasis.

Under immunosuppressed conditions, AMPs restore basal immune function and antigen responsiveness. Studies in mice and chickens show that AMP enhances splenic and peripheral T-lymphocyte proliferation, increases CD4^+^ and CD8^+^ populations, and elevates serum cytokines such as IL-2, IL-6, TNF-α, and IFN-γ [40,86,87]. Mechanistically, these effects depend on activation of the TLR4–MyD88–NF-κB pathway in lymphoid tissues, which triggers transcription of co-stimulatory molecules and survival genes [88]. AMPs also enhance macrophage phagocytic activity and promote controlled NO and cytokine release via IκB degradation and p65 nuclear translocation [47]. In vaccinated animals, AMP supplementation increases IgG titers and antigen-specific antibody responses [89]. Collectively, these actions restore immune vigilance and readiness—an essential foundation for subsequent immune rebalancing during inflammation.

When excessive activation occurs, AMPs counteract inflammation by suppressing multiple signaling cascades. In DSS-induced colitis, AMP administration reduces the expression of TNF-α, IL-1β, and IL-6, accompanied by decreased neutrophil infiltration [68]. At the molecular level, AMPs inhibit phosphorylation of MAPK (ERK, JNK, p38) and NF-κB pathways, thereby dampening the transcription of pro-inflammatory genes [74]. Moreover, AMPs regulate inflammation through epigenetic control: the AMP-induced lncRNA ITSN1-OT1 sequesters phosphorylated STAT2 in intestinal epithelial cells, preventing nuclear translocation and overexpression of interferon-stimulated genes [27]. These actions converge to restrain cytokine storms and facilitate mucosal repair.

AMPs also fine-tune the adaptive immune compartment to prevent chronic inflammation. In DSS-colitic mice, AMP treatment re-establish Th17/Treg balance—reducing Th17-driven IL-17 production while expanding Foxp3^+^ Tregs in mesenteric lymph nodes and spleen [74,90]. This rebalancing is associated with inhibition of the IL-6/STAT3 axis, a key determinant of Th17 differentiation. Consequently, AMPs tilt the immune milieu toward a regulatory, IL-10-rich state that supports long-term tolerance. Additionally, AMPs help sustain mucosal humoral defense by stabilizing secretory IgA levels, enhancing pathogen exclusion without provoking inflammatory overreaction [91]. Through these coordinated actions, AMPs promote an adaptive immune phenotype that resolves inflammation while preserving protective immunity.

Oxidative stress is a major driver of chronic intestinal inflammation, and AMPs mitigate this process through coupled antioxidant and anti-pyroptotic mechanisms. In LPS-challenged macrophages, AMPs suppress NLRP3 inflammasome activation and caspase-1–dependent pyroptosis via the lncRNA GAS5/miR-223 axis, leading to reduced IL-1β and IL-18 release [92]. Complementary studies show inhibition of the SIRT1/NLRP3/caspase-1 pathway in intestinal tissues, further preventing pyroptotic injury [93]. AMPs concurrently elevate superoxide dismutase (SOD) and glutathione peroxidase (GSH-Px) activities while reducing malondialdehyde (MDA) levels, collectively reinforcing the mucosal antioxidant barrier [85]. By integrating redox control with immune resolution, AMPs protect epithelial integrity and support tissue recovery from oxidative-inflammatory stress.

Altogether, AMPs function as context-dependent immunomodulators—enhancing immune competence under suppression, restraining hyperinflammation when overactivated, and sustaining redox balance throughout the mucosa.

### 3.4. Neuromodulatory Actions on Motility and Sensory Pathways

Beyond immunometabolic regulation, AMPs also exert neuromodulatory actions on intestinal motility and sensory signaling. AMPs display bidirectional control on intestinal motility through coordinated modulation of the enteric nervous system and neuro-endocrine mediators. Acting at the interface of the enteric nervous system (ENS), enteroendocrine signaling, and visceral sensory pathways, AMPs normalize transit and alleviate visceral hypersensitivity—two hallmarks of gut functional imbalance. Their effects are best characterized as bidirectional: promoting propulsion under hypomotile or “spleen-deficiency” states while restraining hypersecretory or hypermotile conditions associated with diarrhea.

Experimental models demonstrate that AMPs restore coordinated peristalsis through fine-tuning of serotonergic (5-hydroxytryptamine, 5-HT) and cholinergic circuits in the ENS. In slow-transit constipation, AMP supplementation increased fecal output and water content while normalizing colonic serotonin metabolism—up-regulating tryptophan hydroxylase-1 (TPH-1) and down-regulating serotonin-reuptake transporter (SERT), which together enhance mucosal 5-HT availability [70,94]. These adjustments translate into improved contractile amplitude and transit velocity. Conversely, in secretory diarrhea triggered by tyrosine-kinase inhibitors, AMPs reduce excessive cAMP accumulation and chloride efflux by activating the LKB1/AMPK pathway, which inhibits CFTR-mediated secretion [82]. This dual modulation underscores AMPs’ capacity to recalibrate neurotransmitter-driven motility toward physiological equilibrium.

Beyond neurotransmitter regulation, AMPs integrate microbial and metabolic cues into enteric signaling. By enhancing the production of short-chain fatty acids and tryptophan-derived metabolites, AMPs may influence enterochromaffin-cell activity and vagal afferent communication, thereby stabilizing intestinal motility and visceral comfort. These microbial metabolites—including indole-3-propionic acid and butyrate—can serve as ligands for nuclear receptors such as the AhR and PXR, which modulate mucosal neurotransmitter release and maintain anti-inflammatory tone [73,74]. In parallel, AMP-driven activation of AhR and GLP-1 signaling has been linked to enhanced enteroendocrine peptide secretion, coupling motility regulation with metabolic and stress-response pathways along the gut–brain axis. Collectively, these findings suggest that AMPs harmonize neural, immune, and metabolic signaling networks to sustain intestinal neuro-immune–metabolic homeostasis, providing a mechanistic basis for their therapeutic effects along the gut–brain axis.

**Figure 1:**
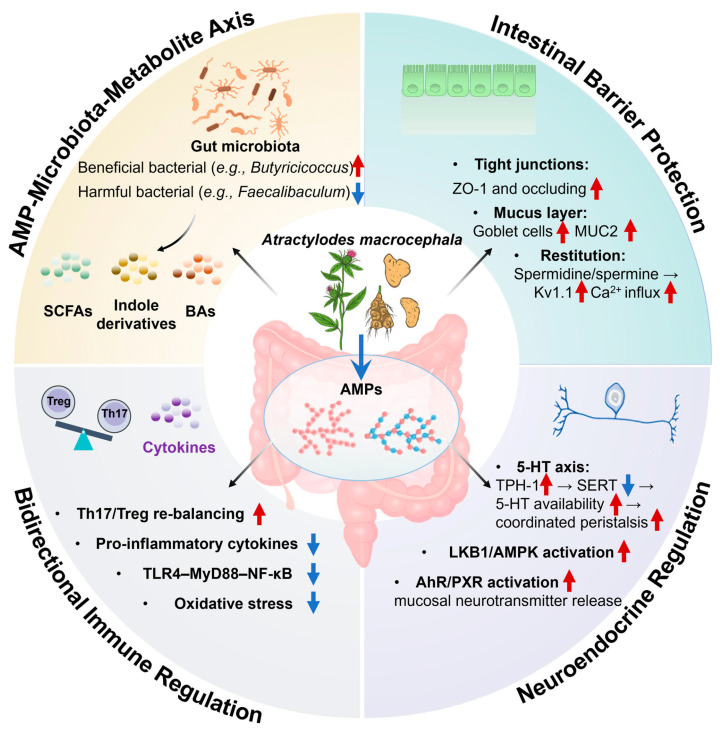
Mechanistic landscape of AMPs in intestinal homeostasis. Red upward arrows denote increases, and blue downward arrows denote decreases.

Overall, AMPs orchestrate a multilevel network involving microbial, epithelial, immune, and neural pathways to sustain gut homeostasis, as illustrated in Figure 1.

## 4. Therapeutic Applications in Intestinal Disorders

Building on the mechanistic findings outlined above, AMPs have been evaluated in a range of intestinal disease models. Notable therapeutic effects have been observed in functional bowel disorders, inflammatory diseases, malignancies, and treatment-induced injury. As summarized in Section 3, these models frequently show convergent responses to AMP treatment, including restoration of microbiota-derived metabolites, reinforcement of epithelial tight junction integrity, modulation of mucosal immune and inflammatory signaling pathways, and neuromodulatory effects on intestinal motility and visceral sensory processing. In Section 4.1, Section 4.2, Section 4.3, Section 4.4 and Section 4.5, disease-specific models, AMP regimens and key outcomes are summarized (Table 2), with only brief reference to these shared mechanisms.

### 4.1. Irritable Bowel Syndrome (IBS)

IBS is a chronic functional bowel disorder characterized by abdominal pain, bloating, and altered stool patterns (diarrhea and/or constipation) in the absence of structural abnormalities [98]. Its pathophysiology is multifactorial, involving gut–brain axis dysregulation, immune activation, visceral hypersensitivity, and gut microbiota imbalance [99]. Given the limited efficacy of conventional pharmacotherapies (antispasmodics, neuromodulators, probiotics) [100], interest has shifted toward safe, microbiota-modulating biopolymers such as AMPs.

Preclinical studies show that AMPs exert bidirectional regulation on intestinal transit. In “spleen-deficiency” diarrhea induced by *Folium sennae*, oral AMP administration significantly improved stool consistency and colon length while reducing diarrhea scores [65]. In functional constipation models analogous to IBS-C, purified AMP fractions (e.g., AC1) restored colonic transit by elevating 5-HT and its biosynthetic enzyme TPH1 while suppressing SERT expression [70,94]. These adjustments rebalanced enteric neurotransmission and supported recovery of motility and sensory thresholds.

Beyond effects on transit, AMPs influence the microbiota–immune–epithelial axis in IBS-like models. AMP administration has been reported to increase fermentative genera such as *Bacteroides*, *Prevotella*, *Odoribacter* and to reduce potentially pro-inflammatory taxa, in parallel with higher fecal SCFA levels and up-regulation of anti-inflammatory cytokines such as IL-10 [94]. In spleen-deficiency diarrhea rats, AMP also expanded CD4^+^CD25^+^Foxp3^+^ Treg cells and increased thymus and spleen indices [65], restoration of immune homeostasis rather than microbiota changes alone. A meta-analysis of functional diarrhea syndromes further confirmed that AMP-based interventions reduced major pro-inflammatory cytokine readouts and MPO, while elevating IL-10. Although IBS lacks the frank inflammation seen in IBD, low-grade mucosal injury and mast-cell activation are common, and in diarrheal IBS-like models AMP treatment reduced mucosal damage and restored goblet cell [65]. By stabilizing barrier function and attenuating sub-inflammatory signaling, AMPs disrupt the cycle of epithelial leakiness and neural hypersensitivity.

Through coordinated modulation of intestinal transit, microbiota composition, mucosal immune tone and barrier integrity, AMPs appear to act on several components of IBS pathophysiology. Preclinical data suggest that these effects can translate into improved bowel function and reduced visceral hypersensitivity in IBS-like models. However, current evidence is limited to heterogeneous animal studies using different AMP preparations and dosing regimens, and dedicated clinical trials in IBS are still lacking.

### 4.2. Inflammatory Bowel Disease (IBD)

IBD, comprising ulcerative colitis and Crohn’s disease, is driven by chronic mucosal inflammation, barrier disruption, and microbial dysbiosis. Conventional anti-inflammatory and immunosuppressive therapies can induce remission in a proportion of patients but are often limited by adverse effects and incomplete mucosal healing [101,102]. Preclinical studies suggest that AMPs may help maintain intestinal homeostasis in IBS through combined effects on immune regulation, epithelial protective, and gut microbiota.

Across several DSS-induced colitis models, AMPs consistently alleviated clinical and histological indices. Oral administration reduced body-weight loss, diarrhea, and hematochezia, preserved colon length, and lowered disease activity index and histopathology scores [90]. Colonic sections from AMP-treated mice showed less epithelial erosion and neutrophil infiltration, with decreased myeloperoxidase activity. A defined AMP fraction (AMP-1) produced similar benefits and was associated with IL-17RA-related signaling and gut microbial changes [103]. In complementary in vitro assays, an AMP-derived fraction (RAMPtp) enhanced the survival and proliferation of intestinal epithelial cells exposed to DSS and helped prevented tight-junction integrity [27].

AMPs also reprogram the mucosal immune response and the gut microbiota in experimental colitis. In DSS-treated mice, AMP intervention reduced key pro-inflammatory cytokines while up-regulating regulatory mediators such as IL-10 and TGF-β and restoring Th17/Treg balance [90]. Purified AMP fraction has been shown to promote Treg differentiation from CD4^+^ T cells and to dampen innate inflammatory activation in macrophage models [47,61]. Other work indicates that AMP- induced shifts in tryptophan metabolizing bacteria and their metabolites contribute to nuclear receptor-mediated anti-inflammatory signaling and epithelial immune quiescence [74]. Structure–activity analyses of several purified AMP fractions (AMAP-1/-2/-3) further suggest that specific rhamnogalacturonan I side chains enhance immunomodulatory potency [26]. In DSS-colitis models, AMPs increased microbial richness and enriched beneficial genera such as *Faecalibaculum*, *Bifidobacterium*, and *Bacteroides*, while reducing potentially harmful taxa such as *Clostridium sensu stricto 1* and *Escherichia Shigella* [68]. Antibiotic depletion and fecal-transplant experiments support a requirement for an intact microbiota for full therapeutic efficacy [74], and AMPs treatment n has been linked to more physiological SCFA, amino acid and bile acid profiles [44,66].

However, most evidence for AMPs in IBD comes from acute DSS models with heterogeneous preparations, and neither chronic/relapsing colitis nor combination therapy with standard IBD drugs has been systematically evaluated.

### 4.3. Colorectal Cancer (CRC)

CRC is one of the most prevalent and lethal malignancies worldwide [104]. Beyond genetic and dietary factors, disruption of intestinal homeostasis and chronic low-grade inflammation play central roles in its pathogenesis [105]. Natural polysaccharides have therefore drawn increasing attention as multifunctional agents that both restore gut equilibrium and exert antitumor effects [106]. Emerging evidence indicates that AMPs suppress CRC progression primarily through immune modulation and microenvironment re-programming, rather than direct cytotoxic activity.

In an orthotopic CRC model, oral administration of a water-extracted AMP fraction (purity ≈ 70%) significantly reduced tumor growth and prolonged survival of MC38 tumor-bearing mice. The antitumor effect was abolished in TLR4-deficient mice, implicating TLR4/MyD88 signaling as an essential mediator of AMP-induced immune activation [96]. In this tumor context, AMP did not directly inhibit tumor-cell proliferation but activated BMDMs, enhancing their phagocytic and migratory activity and increasing the production of TNF-α, IL-6, IFN-λ in BMDMs and in the serum of tumor-bearing mice. These changes were observed in parallel with tumor growth inhibition and are best interpreted as context-dependent amplification of antitumor inflammatory responses, rather than as chronic tissue-damaging inflammation [96]. Complementary findings from a murine in situ colon cancer model confirmed this immune-centric mechanism [107]. AMP administration significantly inhibited tumor growth while up-regulating MHC II and IL-12 expression in dendritic cells and macrophages, accompanied by increased infiltration of CD8^+^ T cells, NK cells, and CD44^+^ lymphocytes, along with augmented IFN-γ secretion. These findings suggest that AMPs convert the CRC microenvironment from immunosuppressive to immunoreactive by activating TLR4-dependent innate sensing and cytotoxic pathways.

Rather than functioning as direct cytotoxins, AMPs appear to act as biological response modifiers that bolster host anti-tumor immunity while helping to preserve intestinal integrity. By promoting macrophage- and T-cell-mediated cytotoxicity and maintaining gut homeostasis, AMPs may enhance immune surveillance against colonic tumor cells and mitigate treatment-related mucosal injury. At present, however, these effects have only been demonstrated in preclinical models, and their interaction with standard chemotherapy, radiotherapy or immunotherapy has not yet been defined.

### 4.4. Chemotherapy-Induced Intestinal Injury

Chemotherapy-induced intestinal mucositis is a frequent and dose-limiting adverse effect of anticancer therapy, characterized by epithelial apoptosis, barrier dysfunction, microbial dysbiosis, and intestinal inflammation [108,109]. These alterations manifest clinically as diarrhea, malabsorption and systemic endotoxemia, compromising both quality of life and treatment adherence. Existing prophylactic approaches, including probiotics and anti-inflammatory agents, provide only partial protection [110]. Although dedicated studies on AMPs in classic mucositis models remain limited, data from several chemotherapy- and toxin-induced injury paradigms suggest that AMPs may confer epithelial and microbiota-mediated protection.

In a cyclophosphamide (CTX)–induced intestinal-injury model, PAM supplementation improved body-weight gain, preserved villus morphology and increased activities of antioxidant enzymes (SOD, GSH-Px), while reducing malondialdehyde accumulation and up-regulating tight-junction-related genes (ZO-1, occludin) [67]. Anti-inflammatory cytokines IL-10 and TGF-β were also increased, indicating concurrent support of epithelial and immune functions under cytotoxic stress. In a pyrotinib-induced diarrhea model, AMP administration ameliorated diarrheal symptoms and restored barrier continuity. These effects were associated with reduced intracellular cAMP, activation of the LKB1/AMPK pathway and inhibition of CFTR-mediated chloride secretion, as well as partial normalization of the gut microbiota, including reversal of drug-induced enrichment of *Clostridium* and *Erysipelotrichi* spp. [82].

In a pirarubicin-treated breast cancer mouse model, AMP supplementation attenuated intestinal ferroptosis and improved mucosal architecture [49]. These effects were accompanied by marked shifts in gut microbial composition, including reduced relative abundances of *Bacteroidaceae*, *Lachnospiraceae*, *Oscillospiraceae* and *Clostridium* spp. and an increase in *Alistipes*, together with partial normalization of metabolites such as glycocholic acid, L-phenylalanine, and palmitoylcarnitine. Antibiotic-mediated depletion of the microbiota abrogated these protective effects, indicating that mitigation of chemotherapy-associated intestinal injury by AMPs is at least partly microbiota dependent.

Taken together, available studies suggest that AMPs mitigate chemotherapy-related intestinal injury in several experimental settings, largely through antioxidant, barrier-preserving and microbiota-modulating actions. However, these data come from a small number of heterogeneous models using different cytotoxic agents (cyclophosphamide, pyrotinib, pirarubicin), and classical mucositis regimens such as 5-fluorouracil, irinotecan or oxaliplatin have not yet been examined. The potential interaction of AMPs with anti-tumour efficacy is also unknown, highlighting the need for standardized studies in established mucositis models, including combination designs with conventional chemotherapeutic regimens, to define the scope and translational relevance of AMP-based protection.

### 4.5. Metabolic and Systemic Disorders Linked to Intestinal Dysfunction

Metabolic disorders, including obesity, insulin resistance, and metabolic dysfunction–associated steatotic liver disease (MASLD), are closely linked to intestinal dysbiosis, barrier defects, and chronic low-grade inflammation [111,112,113]. Recent studies reveal that AMPs exert metabolic benefits primarily by restoring intestinal homeostasis and modulating gut–liver and gut–brain communication.

AMPs modulate the gut microbiota and intestinal immune environment, thereby secondarily improving systemic metabolic regulation. In high-fat diet–induced metabolic disorder models, AMP administration improved insulin sensitivity and lipid handling in parallel with changes in the gut microbiota and intestinal immune milieu. AMPs restored microbial diversity and enriched beneficial taxa involved in SCFA and tryptophan metabolism, accompanied by activation of gut hormone and IL-22–related pathways that support epithelial integrity and downstream hepatic and pancreatic signaling relevant to β-oxidation and glucose control [73]. Transcriptomic analyses further indicate that AMPs can dampen TLR4/MyD88/NF-κB signaling in liver tissue, with reduced hepatic inflammation and oxidative stress [114]. In Western diet-induced metabolic dysfunction-associated steatohepatitis (MASH), AMP treatment alleviated steatosis and hepatocellular injury and was associated with improvements in anxiety- and depression-like behaviors, together with a more balanced gut microbiota–metabolite profiles [97].

AMPs also protect peripheral organs from endotoxin- and stress-related damage. In LPS–induced hepatic injury, attenuated liver damage by limiting inflammasome-related cell death and reducing pro-inflammatory cytokines, while enhancing anti-inflammatory mediators and anti-oxidant enzyme activities [45,92]. Consistent hepatoprotective effects have been reported in hepatic ischemia–reperfusion models, where AMPs were associated with reduced NF-κB activation and oxidative stress [115]. In avian models, AMP similarly alleviated LPS-induced hepatic damage and modulated stress-response pathways [116], suggesting conserved anti-inflammatory and antioxidant functions across species. Together, these data support a role for AMPs in maintaining immunometabolic homeostasis at the gut–liver interface by dampening innate inflammatory programs and preserving redox balance.

**Figure 2:**
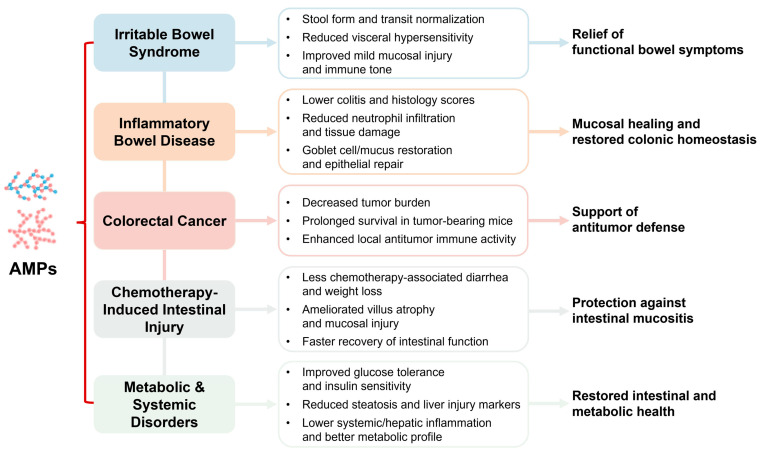
Disease-specific pharmacological effects of AMPs in intestinal and systemic disorders.

Overall, AMPs exert disease-specific protective effects across diverse intestinal and systemic disorders. The main preclinical benefits and therapeutic implications in each indication are summarized in Figure 2.

## 5. Critical Points, Limitations, and Future Perspective

Preclinical studies synthesized in this review indicate that AMPs act primarily within the intestinal lumen on key determinants of gut homeostasis. Across diverse models, AMPs attenuate mucosal inflammation, support epithelial barrier integrity, modulate innate and adaptive immune responses, and reshape gut microbial communities and their metabolic outputs in intestinal, metabolic and systemic disorders linked to dysbiosis and low-grade inflammation. Taken together, current data support the view that AMPs function as gut-targeted immunometabolic modulators that stabilize epithelial barrier function and recalibrate mucosal and systemic immune responses.

Important limitations of the existing evidence must, however, be recognized. Most data derive from preclinical animal models or in vitro systems and thus only partly reflect the complexity and heterogeneity of human disease. Although the studies summarized here largely used AMP-enriched preparations rather than crude extracts, the degree of purification, residual protein or pigment content and potential co-extracted small molecules are not consistently reported, making it difficult to attribute observed effects exclusively to a single, well-defined polysaccharide structure. AMP preparations also differ in plant source, processing, extraction and purification, leading to substantial variation in purity, molecular-weight distribution, monosaccharide composition and linkage patterns. Structural characterization remains incomplete in many reports, and only a minority explicitly relate defined structural features to biological outcomes, limiting robust structure–activity inferences.

Future research on AMPs should prioritize advances in standardization, mechanistic resolution and translational assessment. First, on the chemical and pharmaceutical side, there is a need for AMP preparations with clearly defined ranges of molecular weight, monosaccharide composition, branching and glycosidic linkages, supported by robust analytical fingerprints for reproducible experimentation and quality control. Second, formulation work aimed at improving the stability, bioavailability and colonic delivery of AMPs—including encapsulation, controlled-release systems and combinations with selected probiotic strains or prebiotic substrates—may further enhance local efficacy while preserving safety. Third, mechanistic studies should increasingly adopt integrated multi-omics approaches that combine high-resolution microbiome profiling with metabolomics, proteomics and host transcriptomics to clarify AMP–microbiota–host interactions, identify key taxa and AMP-derived metabolites, and delineate signaling pathways relevant to gut–liver and gut–brain communication. Finally, carefully designed controlled clinical trials using standardized AMP preparations and dosing regimens are needed to establish safety, tolerability and efficacy in indications such as IBS, IBD, MASLD/MASH and chemotherapy-associated intestinal injury. In the nutritional domain, AMPs could be developed as components of functional foods or medical nutrition products, and their integration into personalized nutrition or personalized medicine strategies informed by individual microbiota and metabolomic profiles represents a plausible, albeit still exploratory, direction.

## 6. Conclusions

AMPs constitute a heterogeneous but biologically active group of glycans with reproducible effects on intestinal and immunometabolic homeostasis in experimental systems. Across multiple disease-relevant models, AMPs modulate gut microbial communities and their metabolites, reinforce epithelial barrier function and recalibrate mucosal and systemic immune responses, with additional influences on gut–liver and gut–brain communication. These properties are consistent with their traditional use in chronic gastrointestinal dysfunction and provide a mechanistic rationale for further development of AMPs as gut-targeted nutraceuticals or adjunctive therapies. At the same time, progress is constrained by variability in AMP preparations, incomplete structural characterization, fragmented mechanistic data and the absence of controlled clinical trials. Addressing these limitations through standardized preparations, formulation optimization, multi-omics-informed mechanistic studies and well-designed human investigations will be essential to determine whether the promising preclinical profile of AMPs can be translated into safe, effective and reproducible interventions for intestinal and related systemic disorders.

## Figures and Tables

**Table 1:** Extraction/purification and structural features of AMPs.

Name	MW (kDa)	Monosaccharide Composition	Structural Features	Isolation and Purification Methods	Analytical Methods	Refs.
AMP	23.9	Man, GlcA, Glc, Ara (12: 6: 72: 10)	Typical β-pyranose structure	Hot-water extraction (×3) → EtOH ppt → Sevag deproteinization → organic solvent wash → freeze-drying	GPC-RI-MALS (MW); HPLC–PMP (monosaccharides)	[44]
AMP	8.37	Glc, Gal, Rha, Man (7.36: 1: 3.05: 1.52)	α-(1 → 4) and β-(1 → 4) glycoside linkages	Hot-water extraction → DEAE-Sephacel → EtOH ppt (×3) → dialysis → lyophilization	HPLC (MW); GC (monosaccharides), Limulus assay	[47]
AMAP-1	138	GalA ≫ Gal > Ara > Rha	α-(1 → 4)-GalpA backbone with arabinan/galactan side chains; RG-I like pectic domain	Hot-water extraction → EtOH ppt → dialysis (7 kDa) → DEAE-cellulose → Sepharose 6B → Sephacryl S-300 HR	HPGPC; GC–MS; FT-IR; NMR	[26]
AMAP-2	162	GalA ≫ Gal > Ara > Rha	α-(1 → 4)-GalpA backbone with (1 → 2)/(1 → 2,4)-Rha linkages; arabinan/AG-I side chains	Same as AMAP-1 (major 0.2 M NaCl fraction)	HPGPC; GC–MS; FT-IR; ^1^H/^13^C NMR	[26]
AMAP-3	85	GalA ≫ Ara ≈ Gal > Rha	α-(1 → 4)-linked D-GalpA (~ 73%); minor RG-I regions; C-6 methyl-esterified	Same as AMAP-1 (0.5 M NaCl fraction)	HPGPC; GC–MS; FT-IR; ^1^H/^13^C NMR	[26]
AMP-B	-	Glc, Gal, Man, Ara, Rha (3: 2.5: 1.3: 3.5: 1)	Complex heteropolysaccharide; neutral sugar 50.3%, uronic acid 40.4%, protein 11.5%	Hot-water extraction → EtOH ppt → dialysis → DEAE-cellulose	Elemental analysis; GC (monosaccharides); biochemical assays	[51]
AP	4.25	Ara, Gal, Glc, Man, Gal-UA	Heteropolysaccharide with irregular sheet-like microstructure; FT-IR profile typical of carbohydrates	Hot-water extraction → EtOH ppt → freeze-drying	GPC-RI-MALS; HPLC; FT-IR (4000–400 cm^−1^); SEM	[49]
BZP-1–BZP-5	0.8–90	GulUA, Man, GlcN, Rha, GlcUA, GalUA, GalN, Glc, Gal, Xyl, Ara	α/β-Glcp and α-Galp linkages; morphology, crystallinity, triple-helix altered by stir-frying	Stir-frying → ultrasonic extraction → EtOH ppt → gel filtration (Sepacryl S-100)	HPLC-ELSD; HPLC-PMP; FT-IR; ^1^H/^13^C NMR; SEM; AFM; XRD; Congo red test	[48]
PAM	28.8	Rha, Glc, Man, Xyl, Gal (0.03: 0.25: 0.15: 0.41: 0.15)	β-pyranose structure; coiled morphology (AFM)	Hot-water extraction → EtOH ppt → Sevag → DEAE-52 → Sephacryl S-100	HPGPC; GC–MS; FT-IR; AFM	[28]
PAMK	4.75	Glc, Gal, Ara, Fru, Man (67: 12: 10: 1: 1)	β-Type pyranose configuration	Hot-water extraction → centrifugation → supernatant for GPC-RI-MALS analysis	GPC-RI-MALS; IC for monosaccharides	[45]
RAMPS	109.4	Glc 66.4%, Man, Ara, Gal, Xyl, Rib, Rha	Galp-rich heteropolysaccharide with (1 → 6)/(1 → 3)-linked β-D-Galp	Hot-water decoction → EtOH ppt → Sevag deproteinization → Sephadex A-25 purification	GPC; GC–MS; ^1^H/^13^C NMR	[43]
RAMP	145	Glc, Gal, Ara, Man, Rha, UA (67: 15: 9: 5: 3: 13)	Sulfated heteropolysaccharide with α/β-linkages; pyranose rings.	Ultrasonic-enzymatic extraction → Sevag → EtOH ppt → ultrafiltration → lyophilization	HPGPC; GC-MS; FT-IR; ^1^H/^13^C NMR; SEM; AFM	[53]
RAMP2	4.35	Man, GalA, Glc, Gal, Ara (1.00: 8.58: 27.28: 3.68: 4.99)	β-glucan backbone with α-GalpA and Ara side chains.	Hot-water extraction → EtOH ppt → cation-exchange → DEAE-Cellulose 52→ dialysis	HPSEC-MALLS; HPGPC; PMP-HPLC; FT-IR; ^1^H/^13^C NMR; TEM; SEM	[61]
RAMPS	-	D-Rib, D-Ara, L-Rha, D-Man, D-Glc, D-Gal (1.0: 4.3: 0.1: 5.7: 2.8: 2.2)	Neutral heteropolysaccharide; Glc, Man, Ara as main sugars.	Decoction → EtOH ppt → vacuum-drying	GC–MS; phenol–sulfuric acid (carbohydrate content)	[54]
RAMPtp	1.87	Glc.Man, Rha, Ara, Gal (60.67: 14.99, 10.61, 8.83, 4.90)	β-(1 → 3)/(1 → 6)-linked D-Gal; pyranose form; amorphous morphology	Hot-water extraction → EtOH ppt → papain + Sevag deproteinization → dialysis → freeze-drying	HPLC-PMP; FT-IR; ^1^H/^13^C NMR; SEM; GPC	[25]
PRAM2	19.6	Rha, Xyl, Ara, Glc, Man, Gal (1: 1.3: 1.5: 1.8: 2.1: 3.2)	Neutral heteropolysacwith ~18.1% uronic acid; RG-I-like branching	Defatting → hot-water extraction (×3) → EtOH ppt → Sevag → dialysis→ DEAE-Sepharose Fast Flow → Sephadex G-10	HPSEC (MW); GC; phenol-sulfuric acid; meta-hydroxydiphenyl, Coomassie	[46]
WAM-1	-	Glu, Gal (3: 1)	Rigid chain-like polysaccharide with multi-branching	Degreasing → dialysis → DEAE-Cellulose-52 → Sephadex G-200	GC-MS; NMR; AFM	[42]
WAMPa	4.07	Glc, Ara, Gal (35.9: 7.1: 1.7)	Branched β-D-Glcp backbone with minor α-L-Araf; α/β-linkages	Hot-water extraction → EtOH ppt → Sevag → dialysis → DEAE-52 → Sephadex G-100	SEM; HPGPC; UV; FT-IR; GC-MS; HPLC-PMP; 1D/2D NMR	[55]
Inulin-type AMP	5.84	Fru, Glc, Ara (93: 5: 2)	Inulin-type fructan; linear β-D-Fruf with 1,2,6-linked branches and terminal Glcp	Hot-water extraction → EtOH ppt → dialysis → DEAE-Sepharose FF	HPSEC-MALLS-RID; GC-MS; FT-IR; ^1^H/^13^C NMR	[52]
Inulin-type BZP	2.3	Glc, Fru (α-D-Glcp and β-D-Fruf units; DP = 3–20)	Inulin-type fructan (DP 3–20) including nystose and 1F-fructofranosylnystose	Hot-water extraction → EtOH ppt → enzyme treatment + Sevag→ dialysis → HILIC on XAmide	ESI-MS; HILIC-ELSD; FT-IR; ^1^H/^2^D NMR; HPLC-ELSD	[50]
AMP-ZnONPs	2.7	Ara, Gal, Glc. Xyl, Man, Rib, GalA, GlcA (21.9: 12.3: 34.2: 1: 0.4: 0.9: 0.9: 28.8: 0.7)	Uronic-acid-rich AMP grafted onto ZnO nanoparticles; α/β-configuration; ZnO lattice retained	Alcohol pre-extraction → hot-water extraction → EtOH ppt → Sevag → dialysis→ Sephadex G-100 → grafting to KH550-modified ZnONPs	HPGPC; HPAEC; FT-IR; SEM; TEM-EDS; XRD; XPS; zeta potential	[62]

Abbreviations: BZP, Baizhu (*A. macrocephala*) polysaccharide; Glcp, D-glucopyranosyl residue; Fruf, D-fructofuranosyl residue; DP, degree of polymerization; AMP-ZnONPs, AMP-functionalized zinc oxide nanoparticles; EtOH ppt, ethanol precipitation; DEAE, Diethylaminoethyl cellulose; GPC, gel permeation chromatography; HPGPC, high-performance gel permeation chromatography; HPSEC, high-performance size-exclusion chromatography; HILIC, hydrophilic interaction liquid chromatography; HILIC-ELSD, HILIC with evaporative light-scattering detection; RID, refractive-index detector; ELSD, evaporative light-scattering detector; HPLC, high-performance liquid chromatography; FT-IR, fourier-transform infrared spectroscopy; NMR, nuclear magnetic resonance; SEM, scanning electron microscopy; AFM, atomic force microscopy; XRD, X-ray diffraction; MALS, multi-angle light scattering; PMP, 1-Phenyl-3-methyl-5-pyrazolone derivatization. ESI-MS, electrospray ionization mass spectrometry.

**Table 2:** Summary of preclinical studies on AMPs and their gut-related effects.

Name	Dose and Duration	Experimental Model	Main Gut-Related Effects	Refs.
PAM	0.035–0.05 g/kg, oral, once daily for 10 d (after 10 d modelling)	*C. angustifolia* extract–induced intestinal flora disorder, SD rats	Diarrhea relieved; fecal diversity and community similarity ↑	[28]
AC1	0.5 mL of 0.021 g/mL, oral, once daily for 7 d after constipation induction	Spleen-deficiency constipation, ICR mice (senna + restricted diet/water)	Constipation relieved; microbiota structure shifted toward SCFA/5-HT-favouring taxa and pathways	[94]
PAMK	0.07, 0.14 or 0.28 mg/g, oral, once daily for 7 d after 15 d modelling	Senna leaf + irregular feeding–induced spleen-deficiency constipation, KM mice	Constipation indices improved; 5-HT/brain–gut peptide balance and SCFA–microbiota profile partly normalized	[70]
AMP	0.45 or 0.9 g/kg/day, oral, 7 d after SDD modelling	Spleen-deficiency diarrhea (*Folium sennae*–induced), Sprague–Dawley rats	Diarrhea and colonic damage ↓; Treg responses ↑; *Clostridia* overgrowth ↓, *Bacteroidales*/*Muribaculaceae* ↑; SCFA/bile acid profile improved	[65]
AMP	10, 20 or 40 mg/kg, oral, once daily for 10 d (3 d pretreatment + 7 d treatment)	2.5% DSS-induced ulcerative colitis, C57BL/6J mice	Colitis severity ↓; DSS-induced dysbiosis and fecal/plasma metabolites partly normalized	[44]
AMP	100 mg/kg, oral, once daily for 3 wk (2 wk pretreatment + 1 wk treatment)	3% DSS-induced acute colitis, C57BL/6 mice	Acute colitis and neutrophil infiltration ↓; barrier proteins and SCFA-producing taxa ↑	[68]
PAMK	100, 200 or 400 mg/kg, oral, once daily for 7 d	3% DSS-induced colitis, C57BL/6J mice	Colitis activity and histological injury ↓; barrier integrity ↑; mucosal Th17/Treg balance normalized	[90]
PAMK	100–400 mg/kg, oral, once daily for 7 d; 200 mg/kg used in ABX/FMT	3% DSS-induced colitis in male C57BL/6J mice; antibiotic-depleted microbiota model; fecal microbiota transplantation	DSS colitis attenuated via microbiota-dependent restoration of beneficial taxa, tryptophan metabolites and PXR signaling	[74]
PAMK	400 mg/kg in diet for 28 d	LPS (2 mg/kg i.p., days 24, 26, 28)–induced jejunal injury in goslings	LPS-induced mucosal inflammation ↓; TJ/mucin gene expression ↑; cecal microbiota disruption partly corrected	[69]
PAMK	400 mg/kg in diet for 28 d; LPS 2 mg/kg i.p. on days 24, 26, 28	LPS-induced enteritis, goslings	LPS-induced enteritis and serum cytokines ↓; intestinal morphology and IgA/TJ expression preserved; microbiota disturbance improved	[91]
AP	0.1 or 0.3 g/kg, oral, during chemotherapy	Pirarubicin chemotherapy in breast cancer–bearing C57BL/6 mice	Chemo-induced colitis and epithelial damage ↓; barrier proteins and gut microbiota profile restored	[49]
PAMK	1200 mg/kg (30% PAMK) or 400 mg/kg (95% PAMK) in diet, days 1–28	CTX-induced intestinal injury in Lingnan Yellow chicks (CTX 40 mg/kg i.m., days 19–21)	CTX-induced oxidative and barrier injury in jejunum ↓; cecal richness and composition partly restored	[67]
RAMPS	0.05 g/mouse, oral, once daily for 4 d before each immunisation (two courses, 2-wk interval)	Female ICR mice, RAMPS as adjuvant to FMDV type O vaccination	Intestinal sIgA and mucosal immune cell responses ↑	[95]
PAM	500 mg/kg, i.p., 3×/week for 2 wk starting 7 d after MC38 inoculation	MC38 colorectal cancer xenografts in wild-type and TLR4^−^/^−^ C57BL/6J mice; BMDMs and CRC cell lines (MC38, CT26)	Macrophage anti-tumor activity (phagocytosis, cytokines, NO) ↑; tumor control improved in TLR4-intact but not TLR4^−^/^−^ hosts	[96]
PAMK	700 mg/kg, oral, once daily for 12 wk	Western diet + fructose/glucose + CCl_4_-induced NASH with anxiety/depression-like behaviors, male C57BL/6J mice	NASH-associated dysbiosis corrected (*Firmicutes*/*Bacteroidetes* ratio, key taxa); gut-related carbohydrate and lipid metabolites modulated	[97]
AMP	37.5 or 75 mg/kg, oral, once daily for 6 wk	Ethanol plus high-sugar/high-fat diet (EAHSFD)–induced glycolipid disorder and gut dysbiosis, SD rats	Intestinal barrier function ↑; LPS-driven gut inflammation ↓; dysbiosis and tryptophan metabolites corrected	[73]
PAMPS	50–200 mg/L, 12–24 h	IEC-6 cells (±DFMO/4-AP)	IEC wound closure/migration ↑; DFMO/4-AP–induced impairment reversed	[54]
RAMPtp	5–50 μg/mL, 24 h	3% DSS-injured IPEC-J2 intestinal epithelial cells	DSS-induced IEC apoptosis and cytokine release ↓; TJ protein expression ↑	[27]

Note: ↑, increase or upregulation; ↓, decrease or downregulation.

## Abbreviations

5-HT	5-hydroxytryptamine
AFM	Atomic force microscopy
AMPs	Polysaccharides from *Atractylodes macrocephala* Koidz.
AMP-ZnONPs	AMP-functionalized zinc oxide nanoparticles
AhR	Aryl hydrocarbon receptor
BZP	Baizhu (*A. macrocephala*) polysaccharide
CRC	Colorectal cancer
CTX	Cyclophosphamide
DEAE	Diethylaminoethyl cellulose
DFMO	α-difluoromethylornithine
DSS	Dextran sulfate sodium
EAE	Enzyme-assisted extraction
EGF	Epidermal growth factor
ENS	Enteric nervous system
EtOH ppt	Ethanol precipitation
FT-IR	Fourier-transform infrared spectroscopy
GC-MS	Gas chromatography mass spectrometry
GLP-1	Glucagon-like peptide-1
GSH-Px	Glutathione peroxidase
GPC	Gel permeation chromatography
HPGPC	High-performance gel permeation chromatography
HPLC	High-performance liquid chromatography
IBS	Irritable bowel syndrome
IBD	Inflammatory bowel disease
IECs	Intestinal epithelial cells
LC-MS	Liquid chromatography–mass spectrometry
MAE	Microwave-assisted extraction
MALS	Multi-angle light scattering
MASLD	Metabolic dysfunction-associated steatotic liver disease
MASH	Metabolic dysfunction-associated steatohepatitis
MDA	Malondialdehyde
MUC2	Mucin-2
NLRP3	NLR family pyrin domain containing 3
NMR	Nuclear magnetic resonance
PMP	1-Phenyl-3-methyl-5-pyrazo-lone derivatization
SCFAs	Short-chain fatty acids
PXR	Pregnane X receptor
SEM	Scanning electron microscopy
SERT	Serotonin-reuptake transporter
SOD	Superoxide dismutase
TGF-β1	transforming growth factor-β1
TJs	Tight junctions
Treg	Regulatory T-cell
UAE	Ultrasound-assisted extraction
XRD	X-ray diffraction
ZO-1	Zonula occludens-1

## References

[1] Gao X., Yang C., Feng Z., Liu P., Liu Z. (2025). The signature of the small intestinal epithelial and immune cells in health and diseases. Chin. Med. J..

[2] Gill S.R., Pop M., Deboy R.T., Eckburg P.B., Turnbaugh P.J., Samuel B.S., Gordon J.I., Relman D.A., Fraser-Liggett C.M., Nelson K.E. (2006). Metagenomic analysis of the human distal gut microbiome. Science.

[3] Prorok-Hamon M., Friswell M.K., Alswied A., Roberts C.L., Song F., Flanagan P.K., Knight P., Codling C., Marchesi J.R., Winstanley C. (2014). Colonic mucosa-associated diffusely adherent afaC+ Escherichia coli expressing lpfA and pks are increased in inflammatory bowel disease and colon cancer. Gut.

[4] Quigley E.M. (2013). Gut bacteria in health and disease. Gastroenterol. Hepatol..

[5] Black C.J., Ford A.C. (2020). Global burden of irritable bowel syndrome: Trends, predictions and risk factors. Nat. Rev. Gastroenterol. Hepatol..

[6] Di Vincenzo F., Del Gaudio A., Petito V., Lopetuso L.R., Scaldaferri F. (2024). Gut microbiota, intestinal permeability, and systemic inflammation: A narrative review. Intern. Emerg. Med..

[7] Benede-Ubieto R., Cubero F.J., Nevzorova Y.A. (2024). Breaking the barriers: The role of gut homeostasis in Metabolic-Associated Steatotic Liver Disease (MASLD). Gut Microbes.

[8] Mishra J., Stubbs M., Kuang L., Vara N., Kumar P., Kumar N. (2022). Inflammatory bowel disease therapeutics: A focus on probiotic engineering. Mediat. Inflamm..

[9] Dahiya D., Nigam P.S. (2023). Biotherapy using probiotics as therapeutic agents to restore the gut microbiota to relieve gastrointestinal tract inflammation, IBD, IBS and prevent induction of cancer. Int. J. Mol. Sci..

[10] Zhu B., Zhang Q.L., Hua J.W., Cheng W.L., Qin L.P. (2018). The traditional uses, phytochemistry, and pharmacology of *Atractylodes macrocephala* Koidz.: A review. J. Ethnopharmacol..

[11] Wang J., Wang L., Yang S. (2025). Pharmacological Properties of *Atractylodes macrocephala* Koidz.: A Comprehensive Review. Food Med. Homol..

[12] Wang G., Xie L., Huang Z., Xie J. (2024). Recent advances in polysaccharide biomodification by microbial fermentation: Production, properties, bioactivities, and mechanisms. Crit. Rev. Food Sci. Nutr..

[13] Baky M.H., Salah M., Ezzelarab N., Shao P., Elshahed M.S., Farag M.A. (2024). Insoluble dietary fibers: Structure, metabolism, interactions with human microbiome, and role in gut homeostasis. Crit. Rev. Food Sci. Nutr..

[14] Ferreira S.S., Passos C.P., Madureira P., Vilanova M., Coimbra M.A. (2015). Structure-function relationships of immunostimulatory polysaccharides: A review. Carbohydr. Polym..

[15] Zhao L., Zhang T., Zhang K. (2024). Pharmacological effects of ginseng and ginsenosides on intestinal inflammation and the immune system. Front. Immunol..

[16] Liang H., Tao S., Wang Y., Zhao J., Yan C., Wu Y., Liu N., Qin Y. (2024). Astragalus polysaccharide: Implication for intestinal barrier, anti-inflammation, and animal production. Front. Nutr..

[17] Yue B., Zong G., Tao R., Wei Z., Lu Y. (2022). Crosstalk between traditional Chinese medicine-derived polysaccharides and the gut microbiota: A new perspective to understand traditional Chinese medicine. Phytother. Res..

[18] Zhao T., Wang C., Liu Y., Li B., Shao M., Zhao W., Zhou C. (2025). The role of polysaccharides in immune regulation through gut microbiota: Mechanisms and implications. Front. Immunol..

[19] Kong C., Zhou S., Xu C., Yang J., Chen J., Li C., Yuan Y., Peng Z., Chang G., Yang Y. (2025). Polysaccharides of *Atractylodes macrocephala* Koidz effectively improve the EpCAM(+/−) genotype-related inflammatory bowel diseases via modulating gut microbiota. Int. J. Biol. Macromol..

[20] Luo W., Zhang K., Wang Y., Ye M., Zhang Y., Xu W., Chen L., Li H. (2025). The Rhizome of *Atractylodes macrocephala* Koidz.: A comprehensive review on the traditional Uses, phytochemistry and pharmacology. Chem. Biodivers..

[21] Jeong D., Dong G.Z., Lee H.J., Ryu J.H. (2019). Anti-inflammatory compounds from *Atractylodes macrocephala*. Molecules.

[22] Yang C., Lao Y., Wu F., Su W. (2002). Advances in the study of *Atractylodes macrocephala* Koidz. Zhong Yao Cai.

[23] Lin F., Xu Y., Liu B., Li H., Chen L. (2024). Research progress on extraction, separation, structure, and biological activities of polysaccharides from the genus Atractylodes: A review. Int. J. Biol. Macromol..

[24] Li X., Rao Z., Xie Z., Qi H., Zeng N. (2022). Isolation, structure and bioactivity of polysaccharides from *Atractylodes macrocephala*: A review. J. Ethnopharmacol..

[25] Xu W., Guan R., Shi F., Du A., Hu S. (2017). Structural analysis and immunomodulatory effect of polysaccharide from *Atractylodis macrocephalae* Koidz. on bovine lymphocytes. Carbohydr. Polym..

[26] Cui Y., Li Y., Jiang S., Song A., Fu Z., Dong C., Yao Z., Qiao W. (2020). Isolation, purification, and structural characterization of polysaccharides from *Atractylodis macrocephalae* Rhizoma and their immunostimulatory activity in RAW264.7 cells. Int. J. Biol. Macromol..

[27] Zong X., Xiao X., Kai L., Cheng Y., Fu J., Xu W., Wang Y., Zhao K., Jin M. (2021). *Atractylodis macrocephalae* polysaccharides protect against DSS-induced intestinal injury through a novel lncRNA ITSN1-OT1. Int. J. Biol. Macromol..

[28] Wang R., Zhou G., Wang M., Peng Y., Li X.J.E.b.C. (2014). The metabolism of polysaccharide from *Atractylodes macrocephala* Koidz and its effect on intestinal microflora. Evid. Based Complement. Altern. Med..

[29] Liu X., Lin X., Hong H., Wang J., Tao Y., Huai Y., Pang H., Liu M., Li J., Bo R. (2024). Polysaccharide from *Atractylodes macrocephala* Koidz binding with Zinc Oxide Nanoparticles as a novel mucosal immune adjuvant for H9N2 inactivated vaccine. Int. J. Mol. Sci..

[30] Luo L., Cai J., Zhou Z., Tang W., Xue J., Liu J., Hu H., Yang F. (2022). Polysaccharides from Rhizoma *Atractylodis macrocephalae*: A review on their extraction, purification, structure, and bioactivities. Evid. Based Complement. Altern. Med..

[31] Ji X., Yin M., Nie H., Liu Y. (2020). A review of isolation, chemical properties, and bioactivities of polysaccharides from Bletilla striata. Biomed. Res. Int..

[32] He L., Wu D., Wu X., Cheng J., Li H., Fu L., Wu Q., Wei H., Hu C. (2010). Study on the extraction technology of alkali-soluble polysaccharides from *Atractylodis macrocephalae* Koidz. Med. Plant.

[33] Pu J., Xia B., Hu Y., Zhang H., Chen J., Zhou J., Liang W., Xu P. (2015). Multi-optimization of ultrasonic-assisted enzymatic extraction of *Atratylodes macrocephala* polysaccharides and antioxidants using response surface methodology and desirability function approach. Molecules.

[34] Liang M., Wu Y., Sun J., Zhao Y., Liu L., Zhao R., Wang Y. (2024). Ultrasound-Assisted Extraction of Atractylodes chinensis (DC.) Koidz. Polysaccharides and the Synergistic Antigastric Cancer Effect in Combination with Oxaliplatin. ACS Omega.

[35] Zhang P., Tan J., Wang W., Zhang J., Gong H., Xue H. (2022). Extraction, separation, purification, chemical characterizations, and biological activities of polysaccharides from Chinese herbal medicine: A review. Starch-Stärke.

[36] Zhou P., Xiao W., Wang X., Wu Y., Zhao R., Wang Y. (2022). A comparison study on polysaccharides extracted from Atractylodes chinensis (DC.) Koidz. Using different methods: Structural characterization and anti-SGC-7901 effect of combination with Apatinib. Molecules.

[37] Yang R., Fan H., He B., Ruan Q., Wei B., Han B., Hao X., Maoz I., Kai G. (2023). Current progress of *Atractylodes macrocephala* Koidz.: A review of its biogeography, PAO-ZHI processing, biological activities, biosynthesis pathways, and technology applications. Med. Plant Biol..

[38] Chi Y., Li W., Wen H., Cui X., Cai H., Bi X. (2001). Studies on separation, purification and chemical structure of polysaccharide from *Atractylodes macrocephala*. Zhong Yao Cai.

[39] Wu Q., Li B., Li Y., Liu F., Yang L., Ma Y., Zhang Y., Xu D., Li Y. (2022). Effects of PAMK on lncRNA, miRNA, and mRNA expression profiles of thymic epithelial cells. Funct. Integr. Genom..

[40] Sun W., Meng K., Qi C., Yang X., Wang Y., Fan W., Yan Z., Zhao X., Liu J. (2015). Immune-enhancing activity of polysaccharides isolated from *Atractylodis macrocephalae* Koidz. Carbohydr. Polym..

[41] Tu X.Y., Wang Y.Y., Liu J.W., Li Y.Z., Lu Y., Zhao C.Y., Li Z.S. (2025). Determination of the composition and monosaccharide content of Atractylodes Polysaccharides using pre-column derivatization and a quantitative analysis of multicomponents by a single marker method. J. Chromatogr. Open.

[42] Wu L., Jiang S., Jing Z. (2012). Chromatographic analysis and atomic force microscope observation of polysaccharide extracted from *Atractylodes macrocephala* Koidz. Nat. Prod. Res. Dev..

[43] Fan W., Zhang S., Hao P., Zheng P., Liu J., Zhao X. (2016). Structure characterization of three polysaccharides and a comparative study of their immunomodulatory activities on chicken macrophage. Carbohydr. Polym..

[44] Feng W., Liu J., Tan Y., Ao H., Wang J., Peng C. (2020). Polysaccharides from *Atractylodes macrocephala* Koidz. Ameliorate ulcerative colitis via extensive modification of gut microbiota and host metabolism. Food Res. Int..

[45] Guo S., Li W., Chen F., Yang S., Huang Y., Tian Y., Xu D., Cao N. (2021). Polysaccharide of *Atractylodes macrocephala* Koidz regulates LPS-mediated mouse hepatitis through the TLR4-MyD88-NFkappaB signaling pathway. Int. Immunopharmacol..

[46] Han B., Gao Y., Wang Y., Wang L., Shang Z., Wang S., Pei J. (2016). Protective effect of a polysaccharide from Rhizoma *Atractylodis macrocephalae* on acute liver injury in mice. Int. J. Biol. Macromol..

[47] Ji G., Chen R., Zheng J. (2015). Macrophage activation by polysaccharides from *Atractylodes macrocephala* Koidz through the nuclear factor-kappaB pathway. Pharm. Biol..

[48] Li W., Wang X., Sun X., Zhang Y., Xie J. (2025). Stir-frying duration modulates structural characterization and functional properties of *Atractylodes macrocephala* polysaccharides. Int. J. Biol. Macromol..

[49] Liang Z., Yuan Y., Liang J., Wu Y., Cui J., Gu H., Pi D., Yi Z., Zhou S. (2025). *Atractylodes macrocephala* Koidz. Polysaccharide Alleviates Chemotherapy-Induced Depression-Like Behaviors Through the Gut-Brain Axis. Int. J. Mol. Sci..

[50] Lin Z., Liu Y.F., Qu Y., Shi L.Y., Dou D.Q., Kuang H.X. (2015). Characterisation of oligosaccharides from Baizhu by HILIC-MS. Nat. Prod. Res..

[51] Shan J.J., Tian G.Y. (2003). Studies on physico-chemical properties and hypoglycemic activity of complex polysaccharide AMP-B from *Atractylodes macrocephala* Koidz. Yao Xue Xue Bao.

[52] Tang X., Liu L., Wu Y., Zhao Y., Lu C., Zhao R. (2024). An inulin-type polysaccharide from *Atractylodis macrocephalae* Rhizoma can relieve psoriasis. Int. J. Biol. Macromol..

[53] Yue Y., Li Z., Qian Y., Wang X., Yang H., Su L., Yan S. (2025). Preparation and potential drug delivery applications of *Atractylodes macrocephala* polysaccharide-based nanoparticles. Alex. Eng. J..

[54] Zeng D., Hu C., Li R.L., Lin C.Q., Cai J.Z., Wu T.T., Sui J.J., Lu W.B., Chen W.W. (2018). Polysaccharide extracts of Astragalus membranaceus and *Atractylodes macrocephala* promote intestinal epithelial cell migration by activating the polyamine-mediated K(+) channel. Chin. J. Nat. Med..

[55] Zhuang Y., Huan X.-Y., Sun L., Wang W., Zhou M.-J., Zhao M., Chen P.-D., Yan H., Pang P., Shi X.-Q. (2024). Structural characterization, immunomodulatory effect and immune-mediated antitumor activity of a novel polysaccharide from the rhizome of *Atractylodis macrocephala* Koidz. Results Chem..

[56] Hai C.T., Uyen N.T., Giang D.H., Minh N.T., Duong H.T., Nhat Le B.T., Thanh N.T., Minh T.N., Dat N.T. (2024). Quantitative HPLC-based metabolomics approach for the discrimination of processed rhizomes of *Atractylodes macrocephala*. Curr. Anal. Chem..

[57] Hao Y., Zhang X., Lin X., Yang S., Huang Y., Lai W., Liao X., Liao W., Fu C., Zhang Z. (2024). *The traditional Chinese medicine processing change chemical composition and pharmacological effectiveness: Taking *Atractylodes macrocephala* Koidz. and honey bran-fried *Atractylodes macrocephala* Koidz. as examples. Phytomedicine.

[58] Tang W., Liu D., Nie S.P. (2022). Food glycomics in food science: Recent advances and future perspectives. Curr. Opin. Food Sci..

[59] Merkx D., Westphal Y., van Velzen E., Thakoer K., de Roo N., van Duynhoven J. (2018). Quantification of food polysaccharide mixtures by (1)H NMR. Carbohydr. Polym..

[60] Zeng W., Chen L., Xiao Z., Li Y., Ma J., Ding J., Yang J. (2023). Comparative study on the structural properties and bioactivities of three different molecular weights of lycium barbarum Polysaccharides. Molecules.

[61] Xue W., Gao Y., Li Q., Lu Q., Bian Z., Tang L., Zeng Y., Chen C., Guo W. (2020). Immunomodulatory activity-guided isolation and characterization of a novel polysaccharide from *Atractylodis macrocephalae* Koidz. Int. J. Biol. Macromol..

[62] Bo R., Liu X., Wang J., Wei S., Wu X., Tao Y., Xu S., Liu M., Li J., Pang H. (2022). Polysaccharide from *Atractylodes macrocephala* Koidz binding with zinc oxide nanoparticles: Characterization, immunological effect and mechanism. Front. Nutr..

[63] Yuan S., Li Y., Li J., Xue J.C., Wang Q., Hou X.T., Meng H., Nan J.X., Zhang Q.G. (2022). Traditional Chinese Medicine and Natural Products: Potential Approaches for Inflammatory Bowel Disease. Front. Pharmacol..

[64] Wang X., Li X., Zhang L., An L., Guo L., Huang L., Gao W. (2024). Recent progress in plant-derived polysaccharides with prebiotic potential for intestinal health by targeting gut microbiota: A review. Crit. Rev. Food Sci. Nutr..

[65] Huang S., He H., Li H., Li C., Wang F., Hu Y., Liu Y., Chen L., Chen H. (2025). Modulating the intestinal flora involves the effect of *Atractylodis macrocephalae* Rhizoma polysaccharide on spleen deficiency diarrhea. Int. J. Biol. Macromol..

[66] Hu P., Yan X., Zeng Y., Jiang Z., Liu J., Feng W.W. (2023). An UPLC-MS/MS method for targeted analysis of microbial and host tryptophan metabolism after administration of polysaccharides from *Atractylodes macrocephala* Koidz. in ulcerative colitis mice. J. Pharm. Biomed. Anal..

[67] Lu B., Pan S., He J., Li B., Cao N., Fu X., Liu W., Huang Y., Tian Y., Xu D. (2025). Protective effects of polysaccharide of *Atractylodes macrocephala* Koidz and Jiawei Si-jun-zi Decoction on gut health and immune function in cyclophosphamide-treated chicks. Poult. Sci..

[68] Kai L., Zong X., Jiang Q., Lu Z., Wang F., Wang Y., Wang T., Jin M. (2022). Protective effects of polysaccharides from *Atractylodes macrocephalae* Koidz. against dextran sulfate sodium induced intestinal mucosal injury on mice. Int. J. Biol. Macromol..

[69] Li W., Lu B., Pan S., Bai S., He J., Li B., Cao N., Fu X., Wei J., Chen Y. (2025). Protection of LPS-induced intestinal injury in goslings by polysaccharide of *Atractylodes macrocephala* Koidz based on 16S rRNA and metabolomics analysis. PLoS ONE.

[70] Chen L., Chang X., Wu C., Luo G., Zhang P., Tian W. (2024). Polysaccharide extracted from *Atractylodes macrocephala* improves the spleen deficiency constipation in mice by regulating the gut microbiota to affect the 5-HT synthesis. Neurogastroenterol. Motil..

[71] Schulthess J., Pandey S., Capitani M., Rue-Albrecht K.C., Arnold I., Franchini F., Chomka A., Ilott N.E., Johnston D.G.W., Pires E. (2019). The Short Chain Fatty Acid Butyrate Imprints an Antimicrobial Program in Macrophages. Immunity.

[72] Zhang D., Jian Y.P., Zhang Y.N., Li Y., Gu L.T., Sun H.H., Liu M.D., Zhou H.L., Wang Y.S., Xu Z.X. (2023). Short-chain fatty acids in diseases. Cell Commun. Signal..

[73] He Z., Guo J., Zhang H., Yu J., Zhou Y., Wang Y., Li T., Yan M., Li B., Chen Y. (2023). *Atractylodes macrocephala* Koidz polysaccharide improves glycolipid metabolism disorders through activation of aryl hydrocarbon receptor by gut flora-produced tryptophan metabolites. Int. J. Biol. Macromol..

[74] Zhang Q., Yang M., Liao C., Taha R., Li Q., Abdelmotalab M., Zhao S., Xu Y., Jiang Z., Chu C. (2025). *Atractylodes macrocephala* Koidz polysaccharide ameliorates DSS-induced colitis in mice by regulating the gut microbiota and tryptophan metabolism. Br. J. Pharmacol..

[75] Chen F., Li B., Li W., Chen W., Huang Y., Tian Y., Yang B., Yuan M., Xu D., Cao N. (2023). Polysaccharide of *Atractylodes macrocephala* Koidz alleviate lipopolysaccharide-stimulated liver inflammation injury of goslings through miR-223/NLRP3 axis. Poult. Sci..

[76] Yang P., Qin L., Yu M., Zou Z. (2025). Rhizome of *Atractylodes macrocephala* alleviates spleen-deficiency constipation in rats by modulating gut microbiota and bile acid metabolism. J. Ethnopharmacol..

[77] Neurath M.F., Artis D., Becker C. (2025). The intestinal barrier: A pivotal role in health, inflammation, and cancer. Lancet Gastroenterol. Hepatol..

[78] Chelakkot C., Ghim J., Ryu S.H. (2018). Mechanisms regulating intestinal barrier integrity and its pathological implications. Exp. Mol. Med..

[79] Turner J.R. (2009). Intestinal mucosal barrier function in health and disease. Nat. Rev. Immunol..

[80] Camilleri M. (2019). Leaky gut: Mechanisms, measurement and clinical implications in humans. Gut.

[81] Gao F., Su J., Li J., Gan C., Jin X., Zhou H., Liu X., Yu J., Yan M., Chen S. (2025). Study on the effects of the *Atractylodes macrocephala* koidz-raphanus sativus l herb pair in alleviating senile constipation via the gut microbiota-SCFAs-5-HT axis. Phytomedicine.

[82] Ran P., Jiang F., Pan L., Shu Y., Hu F., Wang Y., Zhao R., Wang W., Mu H., Wang J. (2025). Polysaccharide from *Atractylodes macrocephala* Koidz. alleviates pyrotinib-induced diarrhea through regulating cAMP/LKB1/AMPK/CFTR pathway and restoring gut microbiota and metabolites. Int. J. Biol. Macromol..

[83] Iversen M., Reinert L., Thomsen M., Bagdonaite I., Nandakumar R., Cheshenko N., Prabakaran T., Vakhrushev S., Krzyzowska M., Kratholm S. (2016). An innate antiviral pathway acting before interferons at epithelial surfaces. Nat. Immunol..

[84] Gong S., Zhang B., Sun X., Liang W., Hong L., Zhou X., Li W., Tian Y., Xu D., Wu Z. (2025). Polysaccharides of *Atractylodes macrocephala* Koidz alleviate LPS-induced bursa of fabricius injury in goslings by inhibiting EREG expression. Animals.

[85] Liu C., Wang S., Xiang Z., Xu T., He M., Xue Q., Song H., Gao P., Cong Z. (2022). The chemistry and efficacy benefits of polysaccharides from *Atractylodes macrocephala* Koidz. Front. Pharmacol..

[86] Son Y., Kook S., Lee J. (2017). Glycoproteins and Polysaccharides are the Main Class of Active Constituents Required for Lymphocyte Stimulation and Antigen-Specific Immune Response Induction by Traditional Medicinal Herbal Plants. J. Med. Food.

[87] Liu J., Chen X., Yue C., Hou R., Chen J., Lu Y., Li X., Li R., Liu C., Gao Z. (2015). Effect of selenylation modification on immune-enhancing activity of *Atractylodes macrocephala* polysaccharide. Int. J. Biol. Macromol..

[88] Li B., Li W., Tian Y., Guo S., Huang Y., Xu D., Cao N. (2019). Polysaccharide of *Atractylodes macrocephala* Koidz Enhances Cytokine Secretion by Stimulating the TLR4-MyD88-NF-kappaB Signaling Pathway in the Mouse Spleen. J. Med. Food..

[89] Li W., Guo S., Xu D., Li B., Cao N., Tian Y., Jiang Q. (2018). Polysaccharide of *Atractylodes macrocephala* Koidz (PAMK) Relieves Immunosuppression in Cyclophosphamide-Treated Geese by Maintaining a Humoral and Cellular Immune Balance. Molecules.

[90] Yang M., Zhang Q., Taha R., Abdelmotalab M.I., Wen Q., Yuan Y., Zhao Y., Li Q., Liao C., Huang X. (2022). Polysaccharide from *Atractylodes macrocephala* Koidz. ameliorates DSS-induced colitis in mice by regulating the Th17/Treg cell balance. Front. Immunol..

[91] Li W., Xiang X., Li B., Wang Y., Qian L., Tian Y., Huang Y., Xu D., Cao N. (2021). PAMK Relieves LPS-Induced Enteritis and Improves Intestinal Flora Disorder in Goslings. Evid. Based Complement. Altern. Med..

[92] Chen X., Yang S., Long X., Li W., Li B., Fu C., Zhen C., Xu D., Fu X., Cao N. (2025). Polysaccharide of *Atractylodes macrocephala* Koidz alleviate LPS-induced inflammatory liver injury by reducing pyroptosis of macrophage via regulating LncRNA GAS5/miR-223-3p/NLRP3 axis. Front. Pharmacol..

[93] Deng Q., Lu S., Li Y., Quan H., Li W., Hu X., Wang L., Zhu Q. (2025). Polysaccharide of *Atractylodes macrocephala* Koidz alleviated fructose-induced salt-sensitive hypertension and renal injury via suppressing SIRT1/Nlrp3/caspase-1 pathway in mice. J. Ethnopharmacol..

[94] Yang H., Wu C., Chen L., Chang X., Luo G., Wu K., Tian W. (2023). *A. macrocephala* polysaccharide induces alterations to gut microbiome and serum metabolome in constipated mice. Microb. Pathog..

[95] Xie F., Sakwiwatkul K., Zhang C., Wang Y., Zhai L., Hu S. (2013). *Atractylodis macrocephalae* Koidz. polysaccharides enhance both serum IgG response and gut mucosal immunity. Carbohydr. Polym..

[96] Feng Z., Yang R., Wu L., Tang S., Wei B., Guo L., He L., Feng Y. (2019). *Atractylodes macrocephala* polysaccharides regulate the innate immunity of colorectal cancer cells by modulating the TLR4 signaling pathway. OncoTargets Ther..

[97] Yang J., Ou W., Lin G., Wang Y., Chen D., Zeng Z., Chen Z., Lu X., Wu A., Lin C. (2024). PAMK Ameliorates Non-Alcoholic Steatohepatitis and Associated Anxiety/Depression-like Behaviors Through Restoring Gut Microbiota and Metabolites in Mice. Nutrients.

[98] De Palma G., Lynch M.D., Lu J., Dang V.T., Deng Y., Jury J., Umeh G., Miranda P.M., Pigrau Pastor M., Sidani S. (2017). Transplantation of fecal microbiota from patients with irritable bowel syndrome alters gut function and behavior in recipient mice. Sci. Transl. Med..

[99] Ringel Y., Maharshak N. (2013). Intestinal microbiota and immune function in the pathogenesis of irritable bowel syndrome. Am. J. Physiol.-Gastrointest. Liver Physiol..

[100] Simren M., Tack J. (2018). New treatments and therapeutic targets for IBS and other functional bowel disorders. Nat. Rev. Gastroenterol. Hepatol..

[101] Chang J. (2020). Pathophysiology of Inflammatory Bowel Diseases. N. Engl. J. Med..

[102] Neurath M.F. (2019). Targeting immune cell circuits and trafficking in inflammatory bowel disease. Nat. Immunol..

[103] Mao M., Meng Q., Shentu C., Dai S., Zhu N., Hu C., Wu Y., Yuan X. (2025). *Atractylodes macrocephala* polysaccharides 1 ameliorate ulcerative colitis by regulating gut microbiota and IL-17RA signaling pathway. Food Sci. Hum. Wellness.

[104] Hultcrantz R. (2021). Aspects of colorectal cancer screening, methods, age and gender. J. Intern. Med..

[105] Singh G., Chaudhry Z., Boyadzhyan A., Sasaninia K., Rai V. (2025). Dysbiosis and colorectal cancer: Conducive factors, biological and molecular role, and therapeutic prospectives. Explor. Target Antitumor Ther..

[106] Fan J., Zhu J., Zhu H., Zhang Y., Xu H. (2023). Potential therapeutic target for polysaccharide inhibition of colon cancer progression. Front. Med..

[107] Feng Z., Tang S., Guo L., He L., Yang R. (2019). Polysaccharide of *Atractylodes macrocephala* inhibits the growth of mice in-situ colon cancer HT-29 cell xenograft via activating immune cells. Chin. J. Cancer Biother..

[108] Hong B., Sobue T., Choquette L., Dupuy A., Thompson A., Burleson J., Salner A., Schauer P., Joshi P., Fox E. (2019). Chemotherapy-induced oral mucositis is associated with detrimental bacterial dysbiosis. Microbiome.

[109] van Vliet M.J., Harmsen H.J., de Bont E.S., Tissing W.J. (2010). The role of intestinal microbiota in the development and severity of chemotherapy-induced mucositis. PLoS Pathog..

[110] Peterson D.E., Boers-Doets C.B., Bensadoun R.J., Herrstedt J., Committee E.G. (2015). Management of oral and gastrointestinal mucosal injury: ESMO Clinical Practice Guidelines for diagnosis, treatment, and follow-up. Ann. Oncol..

[111] Tremaroli V., Backhed F. (2012). Functional interactions between the gut microbiota and host metabolism. Nature.

[112] Woting A., Blaut M. (2016). The Intestinal Microbiota in Metabolic Disease. Nutrients.

[113] Albillos A., de Gottardi A., Rescigno M. (2020). The gut-liver axis in liver disease: Pathophysiological basis for therapy. J. Hepatol..

[114] Chen J., Yang S., Luo H., Fu X., Li W., Li B., Fu C., Chen F., Xu D., Cao N. (2024). Polysaccharide of *Atractylodes macrocephala* Koidz alleviates NAFLD-induced hepatic inflammation in mice by modulating the TLR4/MyD88/NF-kappaB pathway. Int. Immunopharmacol..

[115] Jin C., Zhang P.J., Bao C.Q., Gu Y.L., Xu B.H., Li C.W., Li J.P., Bo P., Liu X.N. (2011). Protective effects of *Atractylodes macrocephala* polysaccharide on liver ischemia-reperfusion injury and its possible mechanism in rats. Am. J. Chin. Med..

[116] Zhang B., Hong L., Ke J., Zhong Y., Cao N., Li W., Xu D., Tian Y., Huang Y., Chen W. (2023). Polysaccharide of *Atractylodes macrocephala* Koidz alleviate lipopolysaccharide-induced liver injury in goslings via the p53 and FOXO pathways. Poult. Sci..

